# Repositioning canakinumab for non-small cell lung cancer—important lessons for drug repurposing in oncology

**DOI:** 10.1038/s41416-022-01893-5

**Published:** 2022-06-23

**Authors:** Mark P. Lythgoe, Vinay Prasad

**Affiliations:** 1 Department of Surgery and Cancer, Imperial College London, Hammersmith Hospital, Du Cane Road, London, W12 0HS UK; 2 University of California San Francisco, 550 16th St, 2nd Fl, San Francisco, CA 94158 USA

**Keywords:** Non-small-cell lung cancer, Cancer

## Abstract

Canakinumab is an anti-interleukin-1β monoclonal antibody approved for use in a range of immune-related disorders. During the clinical investigation (CANTOS trial) for prevention of cardiovascular complications, therapy was linked to a reduction in both the occurrence and mortality of lung cancer. This unexpected observation fuelled the rapid initiation of four large clinical trials to evaluate potential anticancer efficacy (in combination with chemotherapy and/or immunotherapy), before fully validating these observations in a dedicated study. The first two trials (CANOPY-1 and 2) have now been reported and have both have failed to meet their primary efficacy endpoints. In this article, we explore the scientific and clinical rationale behind the development of canakinumab in oncology, the repurposing approach utilised and implications this may have for the wider drug repurposing field in the development of new cancer medicines.

On October 25, 2021, Novartis announced the anti-interleukin-1β (IL-1β) drug, canakinumab in non-small cell lung cancer (NSCLC) did not improve the primary endpoints of overall survival (OS) and progression-free survival (PFS) in the CANOPY-1 (NCT03631199) Phase III clinical trial [1]. This is the second negative result, following the first Phase III clinical trial CANOPY-2 (NCT03626545) in March 2021, which also found the compound failed to improve the primary endpoints of OS and PFS in NSCLC [2].

Canakinumab is a monoclonal antibody, which blocks the pro-inflammatory cytokine interleukin-1β and is approved for use by the US Food and Drug Administration (FDA) in the management of a range of adult and paediatric immune-related disorders [1]. Enthusiasm for the product was initially fuelled based on unexpected findings from the CANTOS (Canakinumab Anti-Inflammatory Thrombosis Outcome Study) clinical trial [3]. This was a randomised, double-blind study, placebo-controlled study in over 10,000 participants, and was designed to investigate if canakinumab could prevent recurrent vascular events in patients with a persistent pro-inflammatory response (defined by elevated C-reactive protein levels (CRP)) following myocardial infarction. Overall, CANTOS found canakinumab administration was associated with a reduction of cardiovascular events compared to placebo. But more intriguingly, the treatment was also associated with a significant reduction in the occurrence of fatal and non-fatal lung cancers (no difference in other site-specific cancer was detected) [3]. This effect was most pronounced in the higher-dose group demonstrating a relative hazard reduction of 67% (*P* < 0.0001) for total lung cancer and 77% (*P* = 0.0002) for fatal lung cancer [3].

The early enthusiasm and swift failures so far in clinical trials of canakinumab in NSCLC raise important questions about the validity and utility of drug repurposing as a strategy for developing new cancer medicines. We explore the scientific and clinical rationale behind the development of canakinumab, and implications this may have for drug repurposing within oncology.

Since the time of Virchow, inflammation has been linked to tumorigenesis. Drugs which reduce inflammation (e.g. aspirin) have long intrigued investigators as potential anticancer therapies and are still subject to extensive evaluation today (e.g. Add-Asprin; NCT02804815). Pre-clinical studies demonstrate high levels of the pro-inflammatory cytokine IL-1β, the cytokine target of canakinumab, strongly promote tumour growth, invasiveness and angiogenesis [4]. Furthermore, clinically high levels of inflammatory mediators such as IL-1β and CRP have been demonstrated in several cancer types, including NSCLC, and are frequently associated with detrimental outcomes [5]. Therefore, even prior to the unexpected outcome from the CANTOS trial, therapeutic modulation of the interleukin-1 (IL-1) pathway was under extensive clinical investigation in oncology, across a range of different cancers [6].

Observations from the CANTOS trial led Novartis to launch the CANOPY study programme, including three large-scale randomised Phase III clinical trials (CANOPY-A, CANOPY-1, CANOPY-2) and a Phase II clinical trial (CANOPY-N (NCT03968419)) to investigate the clinical utility of canakinumab as a potential treatment in NSCLC. The first study reported was CANOPY-2, which evaluated canakinumab with docetaxel among 237 patients with locally advanced or metastatic NSCLC who were previously treated with an anti-PD1 or PD-L1 immune checkpoint inhibitor and platinum-based chemotherapy. The addition of canakinumab to docetaxel did not improve PFS or OS (10.5 months in combination vs 11.3 months for monotherapy) and was associated a numerically higher incidence of infections (including fatal infections) [2]. The second reported study CANOPY-1, evaluated canakinumab as first-line therapy for locally advanced or metastatic NSCLC in combination with the anti-PD-L1 drug pembrolizumab and platinum-based doublet chemotherapy and similarly did not meet its primary endpoints of PFS and OS [1]. Ongoing clinical trials include the Phase III clinical trial CANOPY-A investigating canakinumab in the adjuvant setting following surgical lung resection and cisplatin-based chemotherapy (if required), and CANOPY-N investigating canakinumab in the neoadjuvant setting either as monotherapy or in combination with pembrolizumab in patients with operable NSCLC prior to surgery. The CANTOS trial undoubtedly cast light on the potential clinical utility of canakinumab in NSCLC; however, the failure of the CANOPY programme to demonstrate efficacy raises important questions about the validity of drug repurposing within oncology.

Firstly, utilising non-primary outcome data as a rationale for clinical investigation of drug repurposing within oncology has significant limitations. These endpoints are well known for generating spurious results, including both false negatives and positives. In the CANTOS trial, lung cancer was not a formally prespecified endpoint, and although reaching statistical significance, the investigators acknowledge chance may be an unlikely but plausible explanation for their findings [3]. Furthermore, the investigators acknowledge additional factors, including differential surveillance and delayed detection of lung cancer, which had the potential to introduce significant bias.

Second, drugs which have not demonstrated single-agent anticancer activity are problematic to evaluate for repurposing [7]. They cannot be evaluated for efficacy in uncontrolled Phase II studies and must proceed straight to later stages of clinical development, such as Phase III clinical trials, without clearly validating efficacy further. Clinical trials are costly, arduous, and the eligible patient can be a scarce resource. Inappropriate clinical trials use vital resources and infrastructure which may be better utilised elsewhere. Both CANOPY-1 and 2 trials were large international Phase III studies designed to establish canakinumab’s efficacy in combination with other anticancer therapies, while the initial CANTOS trial was a monotherapy study. Smaller Phase II studies could have been utilised initially to establish efficacy and synergy with other anticancer therapies before proceeding to costly Phase III evaluation.

Third, incentives in cancer drug development are so distorted they encourage clinical trials with low promise. The CANOPY programme shows how based on a single signal of promise, a company will launch multiple, costly randomised trials that require sizable patient enrolment (projected recruitment across all four trials is 2526 participants). The decision to launch an adjuvant study prior to the completion of metastatic trials is itself bold and novel. Empirical analyses show that no drug has ever succeeded in adjuvant lung cancer, which did not first succeed in the metastatic setting [8].

Finally, drugs selected for repurposing evaluation must have acceptable adverse effect profiles for cancer patients, particularly when combining with other anticancer therapies which may lead to enhancement of toxicity. The FDA drug label for canakinumab states as a precaution ‘may be associated with an increased risk of serious infection’ [9]. In the CANTOS trial, the major toxicity of canakinumab monotherapy was a significant increase in fatal infection and sepsis compared to placebo, signalling that this may be problematic to combine with other anticancer therapies. Furthermore, significant reductions in both neutrophils and platelets, were also noted in the active treatment arm, which is also a common sequalae from many cytotoxic chemotherapy drugs [3]. In CANOPY-2, canakinumab was combined with the cytotoxic chemotherapy drug docetaxel and was associated with numerically higher incidence of infections, including fatal infections [2]. These results have not been fully reported, but potentially show toxicity from the combination of canakinumab and cytotoxic therapies may be problematic. CANOPY-1 has been reported as ‘no unexpected safety signals were observed with the addition of canakinumab to pembrolizumab plus platinum doublet chemotherapy’, however did report a higher incidence of grade 3 and 4 toxicity in the canakinumab (64.1%)-treated group compared to the placebo (59.3%) arm [10].

New drug development in oncology is a challenging process with high attrition during clinical development. Estimates of the probability of success from Phase 1 to regulatory approval range from 3 to 6% and are notably significantly lower in oncology than in other therapy areas such as cardiology, endocrinology and infectious diseases [11, 12]. The successful repositioning of therapies, such as Bacillus Calmette–Guerin (BCG) as intravesical therapy for treating bladder cancer and thalidomide in multiple myeloma, have demonstrated that drug repurposing in oncology can be highly successful [13]. Furthermore, the integration of biomarkers and/or ‘multi-omics’ into oncology drug repurposing pipelines is likely to lead to further success in the future [14, 15]. Following the intriguing findings from the CANTOS, exploring the repurposing of canakinumab in NSCLC was certainly warranted. However, the development of four clinical trials, in the neoadjuvant, adjuvant and metastatic settings (1st and 2nd/3rd line) in combinations with chemotherapy or chemotherapy and immunotherapy, before fully validating observations from the CANTOS trial has currently proven a leap too far, demonstrating some of the key challenges in drug repurposing within oncology. With patent expiry for canakinumab predicted for 2024, this offers a shortened window for clinical development and product exclusivity, which may have been a contributory factor in driving this development. Novartis remain committed to ‘studying canakinumab in lung cancer’ and are awaiting results for the adjuvant CANOPY-A study, which contains a population with greater similarity to the original CANTOS trial, than those in CANOPY-1 and CANOPY-2 [1].

Lessons from the CANOPY programme show we must revaluate current market incentives for drug repurposing within oncology, which are currently so large, that they can motivate corporations to attempt multiple trials, with substantial patient risk, from even a weak signal of market potential.

## Data Availability

Data sharing not applicable to this article as no datasets were generated or analysed during the current study.
